# The Effect of Inequality and Prosperity on the European Market for Gambling Machines: A Socioeconomic Panel Analysis

**DOI:** 10.1007/s10899-023-10213-2

**Published:** 2023-05-19

**Authors:** Talha Şimşek, Linus Weidner

**Affiliations:** 1 Düsseldorf Institute for Competition Economics (DICE), Heinrich-Heine-University Düsseldorf, Universitätsstr. 1, 40225 Düsseldorf, Germany; 2 Department of Human and Social Sciences, University of Wuppertal, Gaußstr. 20, 42119 Wuppertal, Germany; 3 Institute for Gambling and Society (GLÜG), Ruhr-Universität Bochum, Bochum, Germany

**Keywords:** Gambling, Inequality, Prosperity, Europe, Panel analysis, L83, O52

## Abstract

This study examines the potential influence of prosperity and inequality on gambling participation in Europe. We combined data from the Eurostat database, the Global Wealth Report, and the European Casino Association and estimated fixed effects panel regression models. We show that income inequality has a negative effect on the number of gambling machines that flattens for high values, while wealth inequality has a linear negative effect. Moreover, an increase in the disposable income of the lower quintiles leads to significant increases in the number of gambling machines per country. These findings are important for future researchers who relate any kind of economic variable to gambling as well as for policy makers, as our results suggest that the lower-income groups should be given the most attention with regards to gambling regulation.

## Introduction

While many socioeconomic indicators have been the focus of gambling studies (Barnes et al., 2013; Lutter et al., 2018; Welte et al., 2002), inequality has largely been neglected as an explanatory factor for gambling with only a few exceptions (Barry et al., 2007; Bol et al., 2014; Canale et al., 2017; Pabayo et al., 2023). This omission does not seem to be warranted as studies show that higher (income) inequality leads to more risky behavior in experimental settings (Payne et al., 2017). The idea behind our study is based primarily on a study from Barry et al. (2007, p. 151). They argue that different forms of gambling are likely to be equally attractive for all income groups. In line with Barry et al. (2007, p. 151) we argue that lower-income groups would gamble more if income inequality decreases. These groups have little disposable income and are normally restricted in their ability to gamble. They would be able to act on their gambling inclinations more easily were they to experience an increase in income. We therefore address the question of whether changes in inequality and the disposable income of the lower quintiles are reflected in the number of gambling machines.

Studies show that different forms of gambling are unevenly affected by changes in the economic environment (Barry et al., 2007; Bol et al., 2014; Horváth & Paap, 2012). Horváth and Paap (2012) find that the business cycles affect the relationship between inequality and gambling. Recessions for example seem to affect some forms of gambling more than others. While lotteries are not affected by such economic downturns, the growth in casino revenues seems to stagnate, while revenues for betting games seem to decline (Horváth & Paap, 2012). The few studies that have taken inequality into consideration in the context of gambling (Barry et al., 2007; Bol et al., 2014; Canale et al., 2017), conclude that country-level inequality has variable effects depending on the specific form of gambling that is observed. Canale et al. (2017) also show that the relationship between problem gambling and inequality is dependent on regional differences. We focus on gambling machines in our study since they are the most widespread terrestrial gambling platform and are generally more affordable than other types of gambling such as horse-racing or casino-style games. In the context of our study, this is important because we can only examine the effects of inequality if the gambling opportunity is, in principle, accessible to all people.

Inequality is driven by a multitude of influences such as advances in communication technology (Ali et al., 2019) and general increases in economic complexity (Chu & Hoang, 2020). Inequality itself is a complex construct that entails more than just a comparison of economic resources (Greve, 2021). While social aspects of inequality are certainly interesting and worth studying (Adriaans, 2023; Greve, 2021), most gambling studies as well as the present study focus primarily on economic measures like income inequality. There are two reasons for this. Firstly, income statistics are readily available for most countries; secondly, the data are usually the most reliable. What differentiates our study from most others is the fact that we include a measure of wealth inequality as well. Since gambling demand depends in part on purchasing power, we look at inequality and prosperity within countries. It is also possible that prosperity and inequality are related. However, the direction of this effect has been disputed (Barrilleaux & Davis, 2003).

In the present study we analyze the effects of inequality and prosperity on the European market for gambling machines. We also measure the effect of various income groups on gambling expenditure. The study covers 20 EU countries over a timeframe from 2010-2019. For our statistical models, we use fixed effect panel regressions. We show that income inequality has a negative effect on the number of gambling machines in a country. This relationship follows a u-shaped distribution. In our sample, the demand for gambling machines can be explained primarily by changes in income in the lower-income groups. We find no effect of the general level of prosperity on the market for gambling machines in Europe.

## Review

Bol et al. (2014) also analyzed the effect of inequality on gambling. They find that inequality (measured as Gini coefficient) has a positive effect on lottery expenditure and an inverse u-shaped effect on expenditure on pari-mutuel betting (Bol et al., 2014). The authors attribute their finding primarily to “increasing mobility aspirations, availability of resources in the upper part of the distribution, and status anxiety in the lower part of the distribution”. Pabayo et al. (2023) find similar effects in relation to online gambling of Canadian students. Sociological literature suggests that people participate in gambling to reduce emotional stress (Devereux, 1980) and rid themselves of feelings of deprivation (Callan et al., 2011). These feelings of “falling behind” in society are known to be related to the level of inequality in a country (Hastings, 2019). However, this relationship might not only depend on the absolute level of inequality, but also on whether the present inequality is perceived as legitimate (Haack & Sieweke, 2018; Kuhn, 2019; Willis et al., 2015). Unlike other forms of gambling, active playing time can be particularly high on gambling machines^1^ and this increases the likelihood of players’ interacting with each other. This might affect the subjective perception of inequality. Other studies also show that people do not compare themselves with the whole income or wealth distribution but with other people from the same social strata (Knell & Stix, 2020).

In contrast to the finding from Bol et al. (2014), we expect to see that higher inequality leads to fewer gambling machines in a country. Barry et al. (2007, p. 151) argue that different forms of gambling are likely to be equally attractive for all income groups. In line with them we argue that lower-income groups would gamble more if the income inequality decreased. These groups have little disposable income and are normally restricted in their ability to gamble. They would be able to act on their gambling inclinations more easily were they to experience an increase in income. The Bol et al. (2014) leapfrogging explanation does not hold for the market of gambling machines because winning on a gambling machine does not dramatically change the standard of living of the player. Wealth is especially interesting in the context of inequality as it is distributed even more unequally than income (Piketty, 2015). Overall levels of wealth are also a large contributor towards inequality but have, compared to income, been neglected in research (Pfeffer & Waitkus, 2021).

### H1a:

 Lower levels of wealth inequality and income inequality lead to an increase in the number of gambling machines in a country. This effect to follows a u-shaped distribution.

Inequality however does not explain which group of people in particular has an effect on gambling. We argue that an increased purchasing power of the lower-income groups should lead to more gambling machine play. This is in line with the argument from Barry et al. (2007) that income groups have similar preferences regarding gambling but lower-income groups are restricted by their disposable income.

### H1b:

 More income in the lower quintiles of the income distribution leads to an increase in the number of gambling machines in a country.

Prosperity is most commonly captured by the GDP of a country, mostly as a proxy for gross national income (Bartelmus 2018, p. 25). Based on the assumption that greater prosperity at a country level equals greater purchasing power, we assume that expenditure on gambling is also higher in countries with a higher GDP. Other prominent measures of prosperity are income and wealth, which we also include in our analysis. While net worth i.e. the total sum of assets minus any liabilities would be the better measure, even overall wealth figures are hard to obtain on a global scale. There is considerable evidence that prosperity (GDP) is linked more or less directly to well being. Bartelmus (2018, p. 21) says “prosperity is the materialistic side of being better off”. Other studies find that well being or happiness is related to inequality (Alesina et al., 2004). Happiness or well being is also related to gambling. Some clinical studies show this relationship (Kabasakal, 2015; Oei & Raylu, 2015; Tang & Oei, 2011) and some show it even for recreational gambling (Blackman et al., 2019; Humphreys et al., 2021). We therefore also include a happiness variable as a proxy for the prosperity in a country in a robustness check.

### H2:

 Greater prosperity leads to an increase in the number of gambling machines in a country.

Fiedler et al. (2019) analyze the concentration of gambling expenditure amongst a small subgroup of the gambling population and suggest that further studies should take a closer look at the relationship between gambling and inequality. Canale et al. (2017) conducted a survey of 15-year-old students and examined the results together with region-level data on income inequality and overall wealth. One of their findings is that students in regions with higher income inequality are more likely to be problem gamblers than students in regions with lower income inequality. Moreover, problem and pathological gamblers account for a large share of gambling machines revenues (Fiedler et al., 2019). It is therefore our view that any paper seeking to provide an explanation for the demand for gambling machines on the macro-level must also discuss how the underlying mechanisms (inequality and prosperity in our case) relate to problem gambling. We briefly address this point and potential implications for policymakers in the discussion section.

## Data and Method

To address our research question, we created a new dataset based on multiple sources. Our final dataset contains data on 20 EU countries over a timeframe from 2010-2019. The gambling-related data on the number of gambling machines were taken from the yearly reports of the Gaming Technologies Association. While we would have preferred actual numbers on gambling machine revenue per country, such numbers are not available for many of the European countries. The best available option in this regard is tax revenues which are published by the European Commission for most European countries. Unfortunately, the tax rates on gambling machines vary widely by country and type of gambling machine and the reported numbers are often only published as aggregates. Moreover, the basis of taxation also varies from one country to the next. While some countries tax the total revenue from gambling, others only tax the profits. This renders tax revenue largely useless as a proxy for gambling revenue for the purpose of country comparisons. We had to use the number of gambling machines per country as an alternative measure. The number of gambling machines can be used as a proxy as, it depends in part on demand.

We obtained the data for most explanatory variables and all the controls from the Eurostat database. Many of the relevant numbers are also available in purchasing power parity denominations, which allows accurate inter-country comparisons. The descriptive statistics for all variables are set out in Table 1. Table 2 shows the correlations between the variables.

**Table 1:** Descriptive statistics

Statistic	*N*	Mean	SD	Min	Max
Gambling machines (per thousand inh.)	188	2.62	1.87	0.02	7.66
Gini (share)	188	30.08	4.06	20.90	40.80
Gini wealth (share)	186	69.12	8.65	44.60	90.20
Quintile 1 (share)	188	7.95	1.31	5.10	10.40
Quintile 2 (share)	188	13.35	1.09	11	16
Quintile 3 (share)	188	17.62	0.73	15.20	19.30
Quintile 4 (share)	188	22.94	0.58	21.30	24.60
Quintile 5 (share)	188	38.13	2.91	31.10	47.10
GDP (pc)	188	25,741.52	7,637.83	11,821.76	41,098.84
Median wealth (in thousand USD)	188	57.68	45.60	10.95	195.21
Foreign-born (share)	188	9.58	5.33	0.92	19.75
Unemployment rate (share)	188	8.70	4.54	2.00	26.10
Low education (share)	188	28.34	13.82	11.08	70.40
Leisure expenditure (share)	188	3.21	0.59	2.00	4.80

**Table 2:** Correlation matrix

	Variable	1	2	3	4	5	6	7	8	9	10	11	12	13	14
1	Gambling machines (per 1000 inh.)	1	$-0.00$	$-0.06$	$-0.02$	0.03	$-0.01$	0.07	0.01	0.05	0.09	$-0.05$	0.06	$-0.04$	0.15
2	Gini (share)	$-0.00$	1	$-0.06$	$-0.83$	$-0.88$	$-0.71$	0.16	0.91	$-0.34$	$-0.26$	$-0.08$	0.20	0.04	$-0.24$
3	Gini wealth (share)	$-0.06$	$-0.06$	1	0.07	0.12	0.05	$-0.16$	$-0.06$	0.28	0.09	0.12	$-0.17$	$-0.04$	0.08
4	Quintile 1 (share)	$-0.02$	$-0.83$	0.07	1	0.76	0.54	$-0.29$	$-0.74$	0.31	0.22	0.05	$-0.24$	$-0.04$	0.22
5	Quintile 2 (share)	0.03	$-0.88$	0.12	0.76	1	0.69	$-0.21$	$-0.84$	0.37	0.24	0.05	$-0.19$	$-0.05$	0.25
6	Quintile 3 (share)	$-0.01$	$-0.71$	0.05	0.54	0.69	1	0.09	$-0.80$	0.37	0.30	0.17	$-0.17$	0.01	0.26
7	Quintile 4 (share)	0.07	0.16	$-0.16$	$-0.29$	$-0.21$	0.09	1	0.07	$-0.09$	0.08	0.21	0.21	0.05	$-0.03$
8	Quintile 5 (share)	0.01	0.91	$-0.06$	$-0.74$	$-0.84$	$-0.80$	0.07	1	$-0.36$	$-0.27$	$-0.11$	0.18	0.04	$-0.26$
9	GDP (pc)	0.05	$-0.34$	0.28	0.31	0.37	0.37	$-0.09$	$-0.36$	1	0.59	0.44	$-0.34$	0.04	0.24
10	Median wealth (in 1000 USD)	0.09	$-0.26$	0.09	0.22	0.24	0.30	0.08	$-0.27$	0.59	1	0.41	$-0.04$	0.33	0.15
11	Foreign-born (share)	$-0.05$	$-0.08$	0.12	0.05	0.05	0.17	0.21	$-0.11$	0.44	0.41	1	$-0.06$	0.08	0.22
12	Unemployment rate (share)	0.06	0.20	$-0.17$	$-0.24$	$-0.19$	$-0.17$	0.21	0.18	$-0.34$	$-0.04$	$-0.06$	1	0.14	$-0.14$
13	Low education (share)	$-0.04$	0.04	$-0.04$	$-0.04$	$-0.05$	0.01	0.05	0.04	0.04	0.33	0.08	0.14	1	$-0.07$
14	Leisure expenditure (share)	0.15	$-0.24$	0.08	0.22	0.25	0.26	$-0.03$	$-0.26$	0.24	0.15	0.22	$-0.14$	$-0.07$	1

### Dependent Variable

#### Gambling Machines (Per Thousand Inhabitants)

**Fig. 1:**
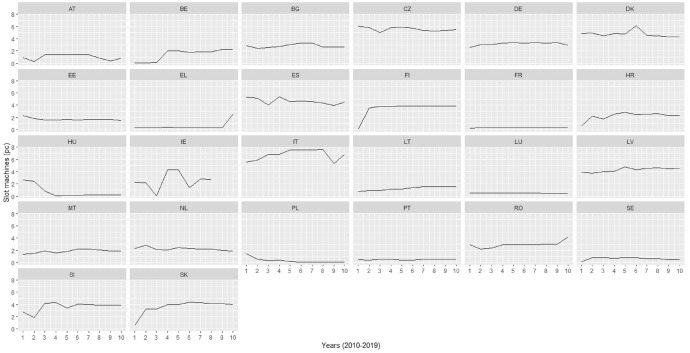
Gambling machines per thousand inhabitants

We obtained the data on the number of gambling machines from the yearly reports of the Gaming Technologies Association. We use the term gambling machines in a broad sense in our study: the variable includes slot machines, video lottery terminals, video gaming machines and electronic table games. The dependent variable contained some severe outliers for some country/year combinations. It is difficult to check whether these more extreme numbers are a result of misreporting by some authorities or gambling providers or if they are indeed accurate. To make sure that these outliers did not affect our data negatively, we dropped them from some of our analyses as a robustness check (see robustness checks in the results section). In Fig. 1 we show the distribution of our dependent variable for all countries in our dataset. For the analysis, we dropped countries with unreasonable changes that might be the result of misreporting or significant changes in the regulatory environment, as well as countries that display no real variation with regards to the number of gambling machines within the timeframe of our analysis. This led to the exclusion of Greece, Ireland, Slovenia, France, Luxembourg, and Finland.

### Explanatory Variables

#### Gini Coefficient^2^

We use two variants of the Gini-coefficient in our study. We took the Gini coefficient based on the equivalized disposable income from the Eurostat database. We also include the Gini coefficient as a squared term in our models to account for the possibility of a non-linear effect. A second variant of Gini is calculated based on the national wealth distribution. We obtained these data from the Global Wealth Report that is issued by Credit Suisse each year. We included this variable because it captures a different aspect of inequality than the income-based Gini coefficient, as the wealth distribution in most countries is much more unequal than the income distribution. These data are based on a combination of official statistics and expert estimates and are therefore less reliable than the data that we use for our income measures. Nevertheless, these reports are valuable in that they give fairly accurate numbers on wealth on a global scale.^3^

#### Income Quintiles

Eurostat divides households into five groups of equal size that are sorted by their equivalized disposable income. The first quintile (quintile 1) is defined as the share of the national disposable income in the lowest income group; the same definition applies accordingly to all other quintiles.^4^

#### GDP Per Capita

We include the GDP on a per capita basis as a measure of national prosperity. The value is also adjusted according to the purchasing power “eliminating differences in price levels between countries”.^5^

#### Median Wealth

To capture the absolute level of wealth in a country, we include the median wealth from the Global Wealth Reports. We argue that median wealth is a better measure to reflect the prosperity of the average person in a country than the mean value, since the mean is heavily distorted by the extreme values at the top of the wealth distribution.

#### Additional Prosperity Measures

For the purpose of further robustness checks, we also collected data on equivalized household income per capita but these data are highly correlated with GDP. We therefore had to include each measure separately in our models. We also use the happiness measure from the European Social Survey as a proxy for prosperity in a further robustness check.

### Controls

#### Unemployment Rate

The rate of unemployment controls for the fact that higher inequality resulting from a larger share of people in the lower part of the income distribution might be affected by unemployment. Studies about gambling show that higher rates of unemployment are associated with higher rates of participation in gambling, but most of the sources are not completely reliable or, with just a few exceptions (Mikesell & Zorn, 1987), provide results only for very specific samples. The idea of falling behind in society that was mentioned in the study by Bol et al. (2014) also relates to unemployment.

#### Migration

We include the measure “foreign-born” to control for inter-country mobility and the effect that non-native citizens might have on the number of gambling machines. Kastirke et al. (2015) and Schulte et al. (2021) also conclude that people with a migration background might be at higher risk of developing problematic gambling behavior. The variable was obtained from the Eurostat database.

#### Education

We know that education has a significant influence on prosperity (Pastor et al., 2018; Teulings & Van Rens, 2008) and inequality (Glomm & Ravikumar, 2003; Hendel et al., 2005; Sylwester, 2002), and we therefore include it as a control. Preference for certain gambling types also depends on the educational background of the player (Hing et al., 2016). We obtained educational data from the Eurostat Database. The variable itself is categorical and based on the international ISCED classification system consisting of 8 educational levels. Eurostat further categorizes these levels into low (less than primary, primary and secondary education or levels 0-2), medium (upper secondary and post-secondary non-tertiary education i.e. levels 3–4) and high education (tertiary education i.e. levels 5–8). For our analysis, we calculate what percentage of each category is represented by the survey population, which consists of people between the ages of 15 and 74 in each country. Based on previous studies we assume that it is primarily the low education group that is likely to bias our results.

#### Leisure Expenditure

We use the consumption expenditure of private households from EUROSTAT. The variable is split into various subcategories. We only take into account the expenditure for services that are related to spare time and culture. We include this variable because gambling expenditures is most likely regarded as a part of that budget from a household’s perspective.

### Estimation Method

To analyze the effect of our predictors on the number of gambling machines in a country, we estimate fixed effects models based on our imbalanced panel data set. All our models use two-way i.e. unit (country) and time (year) fixed effects. The fixing of the country-level variation removes the influence of potentially unobserved policy changes within a country. The time fixed effects remove unobserved factors such as policy changes within Europe. This allows us to estimate the effect that an increase in a predictor variable has on the number of gambling machines in our sample irrespective of a specific time or country. Using the independent and dependent variables defined earlier, we obtain the following fixed effects model:

$$\begin{aligned} \begin{aligned} \hat{Gambling\,machines}_{it}&= {\hat{\beta }}_1\,Gini_{it} +... + {\hat{\beta }}_{8}\,ln(Leisure\,expenditure)_it + {\hat{\alpha }}_i + {\hat{u}}_{it}; \\ i&= Austria, Belgium,\ldots , Slovakia;\,\,\,\,\,\,t = 2010, 2011,\ldots , 2019 \end{aligned} \end{aligned}$$

For all statistical models, we use the natural log of all variables to account for skewness of the variables and diminishing marginal effects. For Gini we include the quadratic term instead to test our first hypothesis. For the wealth-based Gini we also estimated a similar model but the quadratic term has no significant effect and we therefore use the natural log of the base value instead.

## Results

In Table 3 we show seven models. In the main model (1) we include all basic variables and controls. We also include the Gini coefficient as a squared term to account for potential non-linear effects. In the next model (2), we include the wealth-based Gini coefficient instead of the income-based Gini to predict the effect of wealth inequality. The models (3)-(7) each contain one of the quintiles instead of the Gini coefficient. This approach allows us to examine inequality in more detail. All models are based on 188 observations except for the second model, which only has 186 because of two unreasonable values on the Gini wealth variable that were dropped (see footnote 3 on page 9). For those readers interested in specific country-level effects, we also have created a model that includes country dummies (see Table 4 in the appendix).

**Fig. 2:**
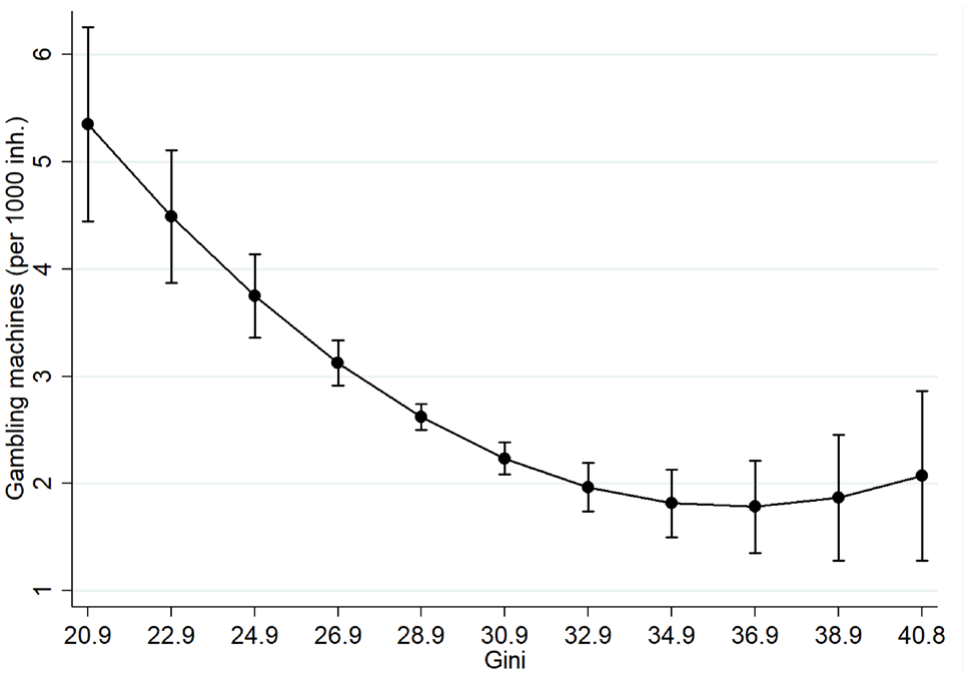
Marginal effect of Gini on gambling machines (95% CIs)

In the main model (1), we see that the Gini coefficient as our measure of income inequality explains a significant share of the number of gambling machines per country. This shows that a decrease in inequality seems to lead to an increase in the number of gambling machines. The squared term is also significant in the model and has a positive effect. This indicates a non-linear relationship between inequality and the number of gambling machines. Figure 2 shows the relationship between the two variables in a graph. Since the effect of the squared term is relatively small in comparison, the effect of inequality on the number of gambling machines per capita is negative and flattens only for high values. In the second model (2) we consider the effect of wealth inequality instead of income inequality. We see that the wealth inequality also has a significant negative effect. An increase of one percentage point in the wealth inequality leads to a decrease of 0.021 gambling machines per thousand inhabitants. In summary, these results confirm H1a except for the expected u-shaped distribution.

**Table 3:** Results of the fixed effects models

	Dependent variable: gambling machines (per thousand inh.)						
	(1)	(2)	(3)	(4)	(5)	(6)	(7)
Gini	$$-1.075^{***}$$						
	(0.227)						
Gini_squared	$$0.015^{***}$$						
	(0.004)						
Gini wealth (ln)		$$-1.452^{**}$$					
		(0.616)					
Quintile 1 (ln)			$$2.281^{**}$$				
			(0.962)				
Quintile 2 (ln)				$$5.312^{***}$$			
				(1.699)			
Quintile 3 (ln)					$$7.445^{***}$$		
					(2.105)		
Quintile 4 (ln)						$6.041^{*}$	
						(3.347)	
Quintile 5 (ln)							$$-6.470^{***}$$
							(1.547)
GDP (pc, ln)	0.810	$-0.232$	$-0.104$	0.370	$-0.173$	$-0.496$	0.044
	(1.135)	(1.226)	(1.218)	(1.216)	(1.191)	(1.234)	(1.175)
Median wealth (ln)	$0.733^{*}$	0.756	0.571	0.562	0.644	0.640	0.659
	(0.438)	(0.485)	(0.475)	(0.469)	(0.466)	(0.480)	(0.459)
Foreign-born (ln)	$$0.794^{***}$$	$$0.740^{***}$$	$$1.024^{***}$$	$$1.009^{***}$$	$$1.160^{***}$$	$$1.078^{***}$$	$$1.074^{***}$$
	(0.247)	(0.282)	(0.258)	(0.255)	(0.256)	(0.263)	(0.249)
Unemployment rate (ln)	$$1.232^{***}$$	$$1.076^{***}$$	$$0.888^{***}$$	$$0.876^{***}$$	$$0.848^{***}$$	$$0.835^{***}$$	$$0.913^{***}$$
	(0.270)	(0.292)	(0.279)	(0.275)	(0.273)	(0.282)	(0.269)
Low education (ln)	$-0.867$	$-0.701$	$-0.807$	$-0.947$	$-1.238$	$-1.283$	$-1.092$
	(0.798)	(0.883)	(0.872)	(0.856)	(0.851)	(0.886)	(0.837)
Leisure expenditure (ln)	$$1.829^{***}$$	$$1.902^{***}$$	$$2.187^{***}$$	$$2.463^{***}$$	$$2.240^{***}$$	$$2.144^{***}$$	$$2.312^{***}$$
	(0.596)	(0.646)	(0.632)	(0.635)	(0.618)	(0.636)	(0.610)
${R}^2$	0.351	0.235	0.228	0.248	0.261	0.217	0.282
Adj. ${R}^2$	0.197	0.056	0.051	0.075	0.090	0.036	0.117
*N* (countries)	20	20	20	20	20	20	20
*N* (country-year)	188	186	188	188	188	188	188
*F* Statistic	10.227^∗∗∗^	6.577^∗∗∗^	6.425^∗∗∗^	7.166^∗∗∗^	7.656^∗∗∗^	6.003^∗∗∗^	8.546^∗∗∗^
	(df = 8; 151)	(df = 7; 150)	(df = 7; 152)	(df = 7; 152)	(df = 7; 152)	(df = 7; 152)	(df = 7; 152)

$$*p<$$0.1; $$**p<$$0.05; $$***p<$$0.01

As the Gini coefficient provides limited information on which group is responsible for the inequality in a country, we use income quintiles to address the provenance of the inequality. In H1b we assume that more income in the lower quintiles leads to more gambling machines in a country. For quintile 1 in model 3 we see a significant positive effect. An increase of one percent in the share of available income in Q1 leads to an increase of 0.02281 gambling machines per thousand inhabitants. This interpretation applies equally to all other quintiles in models 4-7. The effects of quintile 2 in model 4 and quintile 3 in model 5 are significant as well. However, these effects can not be interpreted as straightforwardly as the effect of quintile 1 as we do not know exactly from which quintiles a change in quintile 2 and quintile 3 results. A decline in all other quintiles could lead to an increase in the money supply in quintile 2 or quintile 3 respectively, while for quintile 1 and quintile 5 it is clear that increases always come from the upper or lower quintiles. Together, these models indicate that the supply of gambling machines rises if the income in the lower groups increases. For Q4 we find no significant effect and for quintile 5 (model 7) the effect is in fact negative. This shows that the number of gambling machines per capita decreases if the top earners in society increase their share of the overall income. Both the positive significant coefficient of the lowest income group and the negative significant coefficient of the highest quintile confirm our assumption from H1b.

We can not confirm H2, because the GDP as a measure of prosperity is not significant in all our models (1-7). The same is true for the median wealth variable. We also use other measures of prosperity, which lead to similar results. We explain these robustness checks below.

### Robustness checks

As the dependent variable contained some severe outliers for some country/year combinations, we calculated the percentage change in the number of gambling machines from one year to the next for every country/year combination. This enabled us to cut them from the model based on various exclusion criteria. In Table 5 we see the main model and the reduced models based on our exclusion criteria for outliers. The first model is a full model that is based on a sample of all countries for which we have information on all of the relevant variables (main full). The next model shows the least restrictive case (50% or more deviations) with the model after that being more restrictive (20-% cutoff). The fourth model is the most restrictive, excluding every observation where the number of gambling machines in a country deviates by more than 10% from one year to the next. Finally, we present our main model that we use for all other analyses in this paper (main). Here, we excluded countries that show no variation with regard to the dependent variable or exhibit unreasonable changes from one year to the next (see Data and Method for detailed information). The exclusion of outliers based on our exclusion criteria led to a meaningful loss of observations compared to the full model (main full) with no reasonable benefit in all three cases. The final model in which we manually excluded countries from the dataset is by far the best in terms of overall explanatory power and therefore offers a reasonable balance between losing too many observations and an improved model fit. We therefore only refer to the main model in the discussion section and all other analysis.

The reports of the Gaming Technologies Association contained an asterisk on some of the reported numbers indicating that data collection had changed in some way. Since this might affect the data quality, we created a new binary variable indicating whether the information for that country/year combination might be less reliable.^6^ Table 6 shows that the data quality variable had no significant effects on our model predictions. We did not therefore retain it for further analysis.

As a robustness check we replicate our main model twice using income per capita and once using happiness instead of GDP per capita. These models are shown in Table 7. The results are consistent with the models that include GDP as a measure of prosperity.

To check the validity of our results regarding inequality, we use the 80/20 quintile ratio instead of the Gini coefficient. The S80/S20 income quintile share ratio “refers to the ratio of total equalized disposable income received by the 20% of the country’s population with the highest equalized disposable income (top quintile) to that received by the 20% of the country’s population with the lowest equalized disposable income (lowest quintile)”.^7^ This ratio reflects the inequality in society in a similar way to the Gini coefficient. The model using the 80/20 ratio instead of Gini is set out in Table 8 in the appendix. This model does not differ significantly from our main model, indicating the robustness of our analysis.

## Discussion

We show that an increase in income inequality leads to fewer gambling machines within a country. This is in contrast to the findings on lottery play and pari-mutuel betting from the US (Bol et al., 2014) and online gambling in Canada (Pabayo et al., 2023). For lotteries and gambling machines, this difference makes perfect sense regardless of the country/region: Lotteries attract a different audience and, with higher inequality, the desire to leapfrog to a higher social class increases (Friehe & Mechtel, 2017). We argue that pari-mutuel betting is not directly comparable with lotteries or gambling machines. For pari-mutuel betting, the findings from Bol et al. (2014) suggest a positive non-linear effect of income inequality. We attribute the different findings on gambling and inequality to the fact that gambling markets are unevenly affected by economic changes (Barry et al., 2007; Bol et al., 2014; Horváth & Paap, 2012). Even if gambling machines and pari-mutuel betting are similar in some regards, the contrasting findings might be explained by the different mean inequality in Europe and the U.S. If inequality is high, the share of disposable income is significantly higher in the high-income group which is usually not behind expenditure on gambling machines. On the contrary, we assume in H1b that lower income groups may be a determining factor in the demand for gambling machines. We show this relationship in models 3-7 in Table 3. If the disposable income of the lower income group increases in relation to the other income groups, the number of gambling machines increases (model 3). If the income of the upper income group increases and the income of the lower income groups decreases accordingly, the number of gambling machines decreases as well (model 7). This shows that our findings support each other. Our results show that the assumption from H1b is correct and highlight the added value of a more specific look at the provenance of inequality.

Our results also extend the findings from a study from Italy that shows that the preference for specific forms of gambling varies by income group (Resce et al., 2019) with regards to gambling machines. Furthermore, Barry et al. (2007, p. 151) assume that lower income groups play less due to their lower income. Our study supports their assumption, as we find that when income increases in lower income groups, the demand for gambling machines increases. We also extend these findings by answering the same question for wealth-inequality. However, it might be possible that the changes in wealth are less sustainable for poorer people because of inefficient saving processes (Karlan et al., 2014). Other studies point to the fact that temporary changes in wealth have no effect on private consumption (Lettau & Ludvigson, 2001, 2004). Since our dependent variable reflects the number of gambling machines, it is not possible to reflect short-term changes in demand, resulting from changes in wealth inequality: by the time gambling providers can react to a change in wealth inequality, that inequality might already have decreased again. However, we can not test these assumptions with our data since we can not compare the effect size of the quadratic effect of income inequality with the linear effect of wealth inequality.

Our results show that changes in GDP have no effect on the number of gambling machines when inequality is fixed. Other measures of prosperity such as per capita income, median wealth and overall happiness lead to similar results (see Table 7). An increase in overall prosperity in a country across all income groups benefits everyone, including income groups that are less relevant to demand for gambling machines. This is highlighted in model 6, as the positive effect of the lower income groups offsets the negative effect on gambling expenditure of the highest income group. An increase in prosperity is therefore not a significant predictor for the number of gambling machines.

### Limitations

To reflect a country’s political intervention, we wanted to include a country’s social spending per capita in the regression. However, since this is highly correlated with GDP per capita, we do not include it. The relationship between inequality and gambling might be affected by political interventions, since studies show that welfare policy is related to income inequality (Moene & Wallerstein, 2003; Scruggs & Hayes, 2017). Countries with a more liberal stance towards welfare policy are more likely to see higher levels of inequality than countries with a more social democratic stance (Schneider & Soskice, 2009).

We used the number of gambling machines in a country as a proxy for gambling expenditure. While the number of gambling machines should be a good and reliable alternative, it may not capture certain demand shocks directly as it responds to them more slowly than gambling expenditure. Another point of criticism could be the fact that our dependent variable is skewed between countries. However, the fixed effects model solves this problem because it only considers the variation within a country.

The studies by Freund and Morris (2005, 2006) support the notion that gambling might cause inequality. This, however, indicates that both inequality and gambling might not be exogenous. Bol et al. (2014) have identified this problem before. They tried to resolve it by including a time lag on their measure of inequality (Bol et al. 2014, p. 67). Recently, this procedure has however been criticized as a generally applicable method to address endogeneity problems (Bellemare et al., 2017). According to Bellemare et al. (2017) this statistical solution is only valid if a few assumptions are met. Most importantly, the unobserved variables have to be time-invariant. This assumption is not likely to be true for most large-scale macro data, for which it is difficult to state all the unobserved factors in the first place. Regardless, we do not see a major problem with endogeneity, at least for gambling machines. We find no theoretical explanation for a direct effect of gambling machines on inequality. Expenditure on gambling machines does not directly change the distribution of income among the income groups. We only see the possibility of an indirect effect in the event employment is affected in cases of problematic or pathological gambling. Only then is a change in income reflected in our measure of inequality (Gini). Since the target groups of the different forms of gambling differ, the causal direction of the effect between inequality and gambling is not generalizable. We therefore believe that the specific form of gambling must be considered individually in relation to inequality.

According to Canale et al. (2017), inequality can influence problem gambling behavior, but our model does not allow us to draw any conclusions in this regard. However, we suspect that price elasticity diverges strongly between problem gamblers and casual gamblers. Although we can assume a decrease in gambling with higher inequality, this might have less of an impact on problem gamblers. Consequently, this should be considered, especially in the taxation of gambling machines. Although taxation would lead to fewer gambling machines, the question arises as to what extent problem gambling behavior would be affected. In future studies, the relationship between problem gambling and inequality should be investigated more closely in the context of regulation.

## Conclusion

Based on previous studies (Barry et al., 2007; Bol et al., 2014; Canale et al., 2017; Resce et al., 2019; Pabayo et al., 2023) we argue that national prosperity and income inequality at the country level are useful measures to predict the size of the gambling market at the national level. We test these assumptions with the available data on gambling machines in EU countries.

We found that prosperity has no effect on gambling machines in a country while income inequality has a negative effect that flattens for high values and wealth inequality has a significant negative influence. Demand decreases with increased inequality. Based on this, we took a closer look at inequality by including each of the income quintiles in our analysis. It is particularly interesting to note that when disposable income increases proportionately in the lower quintiles, demand for gambling machines increases. With respect to the negative nonlinear effect of inequality, we differ from previous findings in the literature. It should be noted, however, that comparability is only possible to a limited extent, as the motivation to gamble can differ greatly between the various different forms of gambling. Moreover, the average inequality in Europe is not comparable to the average inequality in the U.S., on which previous studies are based. In summary, our findings suggest that redistribution is an important macroeconomic driver for demand for gambling machines.

Since we only analyzed gambling machines, we encourage the study of further forms of gambling in order to check whether our findings apply to different forms of gambling and across different regions. As we focus on the economic impact of inequality, we use a fairly strict economic definition with the Gini-coefficient. While this allows us to draw on reliable data and keep the research endeavor manageable, it also restricts the scope of our findings with regard to inequality. Further studies should therefore take a closer look at other aspects of inequality in the context of gambling.

Although our study is mainly intended to extend the scientific discussion on inequality and gambling, it is also relevant in terms of policy decisions since we show that the income of the lower income groups in a country drives the demand for gambling machines. Consequently, taxation of gambling machines is more likely to impact the lower income groups. Higher taxation reduces demand, but it is questionable whether this reduces pathological gambling behavior to the same extent.

## Appendix

See Tables 4, 5, 6, 7, 8.

**Table 4:** Individual country effects

	Dependent variable
	Gambling machines (per thousand inh.)
Gini	$$-1.075^{***}$$
	(0.227)
Gini_squared	$$0.015^{***}$$
	(0.004)
GDP (pc, ln)	0.810
	(1.135)
Median wealth (ln)	$0.733^{*}$
	(0.438)
Foreign-born (ln)	$$0.794^{***}$$
	(0.247)
Unemployment rate (ln)	$$1.232^{***}$$
	(0.270)
Low education (ln)	$-0.867$
	(0.798)
Leisure expenditure (ln)	$$1.829^{***}$$
	(0.596)
Belgium	$-0.151$
	(0.629)
Bulgaria	$$6.698^{***}$$
	(1.156)
Czechia	$$6.073^{***}$$
	(0.877)
Germany	$$3.781^{***}$$
	(0.404)
Denmark	$$3.484^{***}$$
	(0.404)
Estonia	$$2.719^{***}$$
	(0.715)
Spain	$$4.072^{***}$$
	(0.776)
Croatia	$$2.758^{***}$$
	(0.717)
Hungary	$$2.292^{***}$$
	(0.753)
Italy	$$7.376^{***}$$
	(0.780)
Lithuania	$$3.277^{***}$$
	(0.900)
Latvia	$$4.726^{***}$$
	(0.868)
Malta	$$2.204^{***}$$
	(0.706)
Netherlands	$$1.321^{***}$$
	(0.429)
Poland	$$2.988^{***}$$
	(0.931)
Portugal	$$2.519^{***}$$
	(0.893)
Romania	$$7.588^{***}$$
	(1.012)
Sweden	$$-1.044^{***}$$
	(0.250)
Slovakia	$$2.863^{***}$$
	(0.786)
${R}^2$	0.955
Adj. ${R}^2$	0.944
*N*	188
*F* Statistic	79.089^∗∗∗^ (df = 31; 210)

$${*}$$ $$p<$$0.1; $${**}$$ $$p<$$0.05; $${***}$$ $$p<$$0.01

Austria is the reference category and therefore excluded from the model

**Table 5:** Removal of outliers based on the dependent variable at 50%, 20% and 10% deviation

	Dependent variable				
	Gambling machines (per thousand inh.				
	(Main full)	(50%)	(20%)	(10%)	(Main)
Gini	$$-0.807^{***}$$	$$-0.494^{**}$$	$$-0.640^{***}$$	$$-0.662^{***}$$	$$-1.075^{***}$$
	(0.270)	(0.207)	(0.186)	(0.207)	(0.227)
Gini_squared	$$0.011^{**}$$	$$0.007^{**}$$	$$0.010^{***}$$	$$0.010^{***}$$	$$0.015^{***}$$
	(0.004)	(0.003)	(0.003)	(0.003)	(0.004)
GDP (pc, ln)	$-0.879$	1.144	0.868	0.150	0.810
	(1.075)	(0.946)	(0.870)	(0.998)	(1.135)
Median wealth (ln)	0.337	0.309	0.513	$$0.871^{**}$$	$$0.733^{*}$$
	(0.472)	(0.356)	(0.337)	(0.399)	(0.438)
Foreign-born (ln)	$$0.962^{***}$$	0.204	$$-0.033$$	$$-0.321$$	$$0.794^{***}$$
	(0.298)	(0.271)	(0.266)	(0.333)	(0.247)
Unemployment rate (ln)	$$0.619^{**}$$	$$0.657^{***}$$	$$0.769^{***}$$	$$0.878^{***}$$	$$1.232^{***}$$
	(0.290)	(0.238)	(0.216)	(0.243)	(0.270)
Low education (ln)	$$-1.901^{**}$$	$$-0.784$$	$$-0.761$$	$$-0.700$$	$$-0.867$$
	(0.884)	(0.639)	(0.571)	(0.685)	(0.798)
Leisure expenditure (ln)	$$1.181^{*}$$	0.491	0.116	0.065	$$1.829^{***}$$
	(0.602)	(0.488)	(0.467)	(0.574)	(0.596)
${R}^2$	0.180	0.089	0.134	0.161	0.351
Adj. ${R}^2$	0.010	$-0.142$	$-0.132$	$-0.170$	0.197
*N* (countries)	26	26	26	26	20
*N* (country-year)	246	204	176	146	188
*F* Statistic	5.553^∗∗∗^	1.968^∗^	2.582^∗∗^	2.486^∗∗^	10.227^∗∗∗^
	(df = 8; 203)	(df = 8; 162)	(df = 8; 134)	(df = 8; 104)	(df = 8; 151)

$${*}$$ $$p<$$0.1; $${**}$$ $$p<$$0.05; $${***}$$ $$p<$$0.01

**Table 6:** Data quality

	Dependent variable	
	Gambling machines (per thousand inh.)	
	(Main)	(Quality)
Gini	$$-1.075^{***}$$	$$-1.044^{***}$$
	(0.227)	(0.226)
Gini_squared	$$0.015^{***}$$	$$0.014^{***}$$
	(0.004)	(0.004)
GDP (pc, ln)	0.810	0.676
	(1.135)	(1.133)
Median wealth (ln)	$0.733^{*}$	$0.755^{*}$
	(0.438)	(0.436)
Foreign-born (ln)	$$0.794^{***}$$	$$0.739^{***}$$
	(0.247)	(0.248)
Unemployment rate (ln)	$$1.232^{***}$$	$$1.245^{***}$$
	(0.270)	(0.268)
Low education (ln)	$-0.867$	$-0.803$
	(0.798)	(0.795)
Leisure expenditure (ln)	$$1.829^{***}$$	$$1.787^{***}$$
	(0.596)	(0.593)
Data quality		0.271
		(0.169)
${R}^2$	0.351	0.362
Adj. ${R}^2$	0.197	0.205
*N* (countries)	20	20
*N* (country-year)	188	188
*F* Statistic	10.227^∗∗∗^ (df = 8; 151)	9.472^∗∗∗^ (df = 9; 150)

$${*}$$ $$p<$$0.1; $${**}$$ $$p<$$0.05; $${***}$$ $$p<$$0.01

**Table 7:** Replication of all models with income and happiness instead of GDP

	Dependent variable		
	Gambling machines (per thousand inh.)		
	(Main)	(Income)	(Happiness)
GDP (pc, ln)	0.810		
	(1.135)		
Income (pc, ln)		0.685	
		(1.069)	
Happiness (ln)			$-0.327$
			(2.416)
Gini	$$-1.075^{***}$$	$$-1.102^{***}$$	$$-0.959^{***}$$
	(0.227)	(0.251)	(0.287)
Gini_squared	$$0.015^{***}$$	$$0.015^{***}$$	$$0.013^{***}$$
	(0.004)	(0.004)	(0.005)
Median wealth (ln)	$$0.733^{*}$$	0.743	$$1.032^{**}$$
	(0.438)	(0.458)	(0.512)
Foreign-born (ln)	$$0.794^{***}$$	$$0.837^{***}$$	$$1.128^{***}$$
	(0.247)	(0.275)	(0.408)
Unemployment rate (ln)	$$1.232^{***}$$	$$1.230^{***}$$	$$1.424^{***}$$
	(0.270)	(0.267)	(0.314)
Low education (ln)	$-0.867$	$-1.021$	$-0.643$
	(0.798)	(0.803)	(0.924)
Leisure expenditure (ln)	$$1.829^{***}$$	$$1.858^{***}$$	$$3.074^{***}$$
	(0.596)	(0.640)	(0.713)
${R}^2$	0.351	0.360	0.457
Adj. ${R}^2$	0.197	0.200	0.274
*N* (countries)	20	19	18
*N* (country-year)	188	176	132
*F* Statistic	10.227^∗∗∗^	9.851^∗∗∗^	10.311^∗∗∗^
	(df = 8; 151)	(df = 8; 140)	(df = 8; 98)

$${*}$$ $$p<$$0.1; $${**}$$ $$p<$$0.05; $${***}$$ $$p<$$0.01

**Table 8:** Replication of the main model with S80/S20 ratio instead of Gini

	Dependent variable	
	Gambling machines (per thousand inh.)	
	(Main)	(Ratio)
Gini	$$-1.075^{***}$$	
	(0.227)	
Gini_squared	$$0.015^{***}$$	
	(0.004)	
S80/S20 ratio		$$-2.353^{***}$$
		(0.601)
S80/S20 ratio_squared		$$0.167^{***}$$
		(0.048)
GDP (pc, ln)	0.810	0.424
	(1.135)	(1.183)
Median wealth (ln)	$0.733^{*}$	0.621
	(0.438)	(0.458)
Foreign-born (ln)	$$0.794^{***}$$	$$0.938^{***}$$
	(0.247)	(0.252)
Unemployment rate (ln)	$$1.232^{***}$$	$$1.052^{***}$$
	(0.270)	(0.275)
Low education (ln)	$-0.867$	$-0.755$
	(0.798)	(0.837)
Leisure expenditure (ln)	$$1.829^{***}$$	$$2.030^{***}$$
	(0.596)	(0.617)
${R}^2$	0.351	0.290
Adj. ${R}^2$	0.197	0.121
*N* (countries)	20	20
*N* (country-year)	188	188
*F* Statistic (df = 8; 151)	10.227^∗∗∗^	7.709^∗∗∗^

$${*}$$ $$p<$$0.1; $${**}$$ $$p<$$0.05; $${***}$$ $$p<$$0.01

## Footnotes

1 For an analysis of the effect of a reduction in the amount of slot machine gambling see Hansen and Rossow (2012).

2 https://ec.europa.eu/eurostat/statistics-explained/index.php?title=EU_statistics_on_income_and_living_conditions_(EU-SILC)_methodology_-_distribution_of_income#Description.

3 For Denmark we identified two unreasonable values in the years 2011 and 2013 with a Gini-coefficient >1. These values have been removed for the purpose of our analysis

4 https://ec.europa.eu/eurostat/statistics-explained/index.php?title=EU_statistics_on_income_and_living_conditions_(EU-SILC)_methodology_-_distribution_of_income#Descriptionhttps://ec.europa.eu/eurostat/statistics-explained/index.php?title=EU_statistics_on_income_and_living_conditions_(EU-SILC)_methodology_-_definition_of_dimensions#Income_quantilehttps://ec.europa.eu/eurostat/statistics-explained/index.php?title=EU_statistics_on_income_and_living_conditions_(EU-SILC)_methodology_%E2%80%93_concepts_and_contents#Income_quantile.

5 https://ec.europa.eu/eurostat/databrowser/view/tec00001/default/table?lang=en.

6 There was no separate report for the year 2014 available but the numbers for 2014 are also reported in the next year’s report, but without information about the data quality. Since we have no information about the data quality for the year 2014, we assume that this number might be unreliable for every country that had any less reliable data in any other year.

7 https://ec.europa.eu/eurostat/statistics-explained/index.php?title=EU_statistics_on_income_and_living_conditions_(EU-SILC)_methodology_-_distribution_of_income#Description.
